# Short-term duration of diabetic retinopathy as a predictor for development of diabetic kidney disease

**DOI:** 10.2478/jtim-2022-0074

**Published:** 2023-12-20

**Authors:** Jiayu Duan, Dongwei Liu, Zihao Zhao, Lulu Liang, Shaokang Pan, Fei Tian, Pei Yu, Guangpu Li, Zhangsuo Liu

**Affiliations:** Research Institute of Nephrology, Zhengzhou University, Zhengzhou 450052, Henan Province, China; TCM-Integrated Department of Nephrology, The First Affiliated Hospital of Zhengzhou University, Zhengzhou 450052, Henan Province, China; Henan Province Research Center for Kidney Disease, Zhengzhou 450052, Henan Province, China

**Keywords:** diabetic kidney disease, diabetic retinopathy, Mendelian randomization, causal effect

## Abstract

**Background:**

Diabetic retinopathy (DR) is a risk factor for diabetic kidney disease (DKD). Whether the duration, especially the short-term duration, of DR is associated with the development and progression of DKD remains unclear.

**Materials and Methods:**

A retrospective study and two-sample Mendelian randomization (MR) analysis were conducted. Kidney disease was defined by the urinary albumin-to-creatinine ratio (ACR) and the estimated glomerular filtration rate (eGFR). DR was diagnosed by an expert ophthalmologist by using a digital fundus camera. Binary and ordinal logistic regression analyses were performed. A restricted cubic spline was utilized to detect nonlinear associations. Summary statistics for DR- and DKD-associated single-nuclear polymorphisms (SNPs) were extracted from the FinnGen and the UK Biobank consortia.

**Results:**

A total of 2674 patients with type 2 diabetes mellitus (T2DM) and type 2 diabetic kidney disease (T2DKD) were included. The prevalence and mean duration of DR increased with elevation of ACR and decline in eGFR. Renal function was significantly reduced in patients with DR in the fifth year of life. Binary and ordinal logistic regression showed that each 1-year increase in DR duration was associated with a 19% risk increase in the development of DKD, 16% in the elevation of ACR, and 21% in the decline of renal function. MR estimates indicated that DR was causally associated with DKD development, with an odds ratio of 2.89.

**Conclusions:**

DR and the duration of DR were independent risk factors for the development and progression of DKD. The short-term duration of DR may be associated with DKD development. DR had a statistically significant effect on DKD.

## Introduction

Diabetic kidney disease (DKD) is one of the most serious microvascular complications of diabetes and the leading cause of end-stage renal disease (ESRD) worldwide.^[1]^ It is important to predict the course of renal impairment because the decline in renal function and response to treatment may differ among patients.^[2]^ However, unselective screening for DKD is expensive, invasive, and difficult to establish in developing countries.^[3, 4, 5, 6]^

In patients with diabetes, prolonged hyperglycemia, hypertension, and oxidative stress contribute to vascular remodeling and vessel dilation, resulting in renal impairment and other microvascular diseases such as diabetic retinopathy (DR).^[7, 8, 9]^ Previous studies have also demonstrated concordance between DKD and DR owing to their mutual mechanisms.^[10]^ Ophthalmoscopy is an inexpensive microvascular examination that can be routinely performed in community screening programs for diabetes complications.

DR has been reported as a valuable predictor of DKD in type 1 diabetes and a risk factor for albuminuria and decline in estimated glomerular filtration rate (eGFR) in patients with type 2 diabetes.^[10, 11, 12]^ Hsieh and Hsieh^[13]^ indicated that remission of microalbuminuria could be a protective factor against the development of DR. These findings suggest that DR and DKD are strongly associated. However, few studies have investigated the association between DR duration, especially its short-term duration, and DKD development. In addition, many previous reports were observational studies with relatively small sample sizes, which made it difficult to investigate the causal effects between DR and DKD.

The Mendelian randomization (MR) approach enables the investigation of potential causal relationships between a risk factor (DR) and an outcome (DKD) by utilizing ideal instrumental variables (IV) that are always provided by genetic data, particularly DNA. Single-nuclear polymorphisms (SNPs) are unique to each individual and are not affected by potential confounders once the individual is born. Genome-wide association studies have provided abundant genetic data resources for magnetic resonance (MR) analysis. Hence, herein, we conducted a retrospective study to investigate the association between the short-term duration of DR and the development of DKD, and a two-sample MR analysis to explore the potential causal relationship between DR and DKD.

## Materials and methods

### Study population

The inclusion criteria for the study participants were as follows: (1) patients who had at least one inpatient encounter with the hospital and (2) patients diagnosed with type 2 diabetes mellitus (T2DM) or T2DM with complications. Exclusion criteria were (1) diagnosis of type 1 diabetes or secondary diabetes (e.g., chronic pancreatitis or pancreatectomy), (2) active malignancy or immune disorders, (3) pregnancy, (4) hyperglycemia caused by medications, (5) need for renal replacement therapy, and (6) incomplete results of blood or urinary tests (i.e., test of kidney function or 24-h urinary albumin excretion).

**Figure 1:**
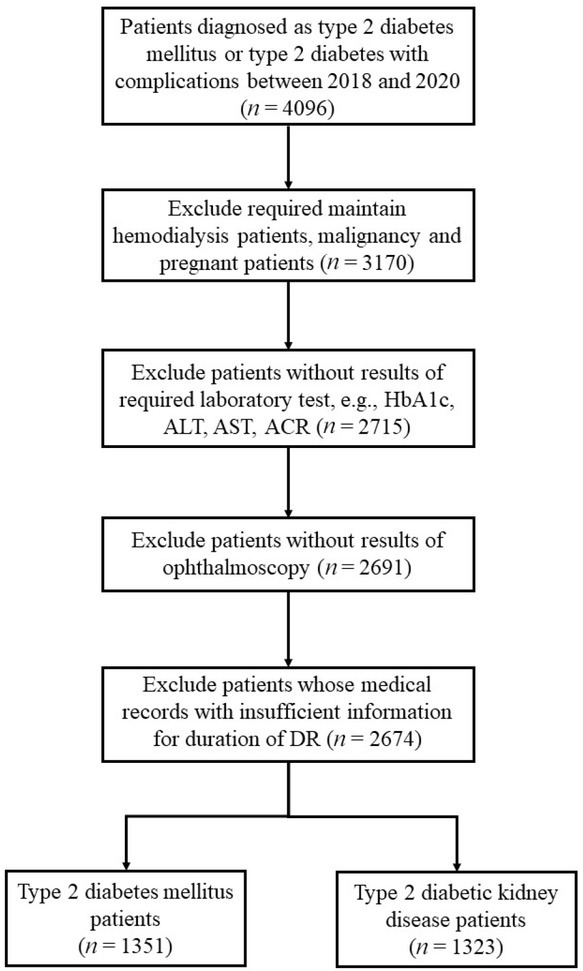
Flow chart of patients’ step-by-step screening.

Patients who met the inclusion criteria from January 1, 2018 to December 31, 2020 were included (*N* = 2674; Figure 1). Data were obtained from preexisting electronic medical records (EMRs) based on the hospital information system. Informed consent was not obtained because this hospital has an opt-in policy for collecting data using EMRs for research purposes. This research complied with all the relevant national regulations and institutional policies, in accordance with the tenets of the Declaration of Helsinki, and was approved by the review board of the hospital (No. KY-2018-LW-001).

### Measurements and definitions

The following information of study participants was collected from their EMRs: demographic information (age, sex, body mass index [BMI]); blood pressure; duration of diabetes and DR; use of angiotensin-converting enzyme inhibitors (ACEIs), angiotensin receptor blockers (ARBs), and sodium–glucose cotransporter 2 (SGLT2) inhibitors; and laboratory test results (serum and urinary).

Hypertension was defined as (1) systolic blood pressure (SBP) ≥140 mmHg and/or diastolic blood pressure (DBP) ≥90 mmHg, (2) self-reported previous diagnosis of hypertension, and (3) use of antihypertensive medications in the past 2 weeks. DR was diagnosed by expert ophthalmologists using a digital fundus camera (CR-2; Canon, Tokyo, Japan). The DR images were further graded according to the international clinical DR severity scales from the Global Diabetic Retinopathy Project Group.^[14]^ DR severity was categorized into no DR (no DR), nonproliferative DR (NPDR), and proliferative DR (PDR). DKD was defined as diabetes with renal impairment, including albuminuria and reduced eGFR. Albuminuria was defined as a urinary albumin/creatinine ratio (ACR) >30 mg/g. Reduced eGFR was defined as a value less than 60 mL/min/1.73 m^2^ calculated by the CKD-EPI 2009 equation. ^[15]^ Dyslipidemia was defined as (1) the presence of abnormal serum lipid concentrations, according to the Chinese guidelines on prevention and treatment of dyslipidemia in adults,^[16]^ (2) self-reported previous diagnosis of dyslipidemia, and (3) use of anti-dyslipidemia medications during the last 2 weeks. Hyperuricemia was defined as a serum uric acid concentration of >422 μmol/L for men and >363 μmol/L for women.

### Statistical analysis

All statistical analyses were performed using R software (version 4.0.2, https://www.r-project.org/) and GraphPad Prism 8 (GraphPad Software, Inc., La Jolla, CA, USA) for Windows. A *P*-value <0.05 was considered statistically significant. Data are expressed as mean ± standard deviation (SD), median with interquartile range (IQR), or frequency with percentage, as appropriate. Intergroup comparisons were performed using the Pearson Chi-square test, Student’s *t*-test, Mann–Whitney U test, and Wilcoxon test for categorical and continuous variables, as appropriate.

Both binary and ordinal logistic regression analyses were conducted to explore the association between the development/duration of DR and indicators of renal damage. Crude and multivariable adjusted odds ratios (ORs) with 95% confidence intervals (CIs) were also calculated. The reference values of covariates in the multivariable regression model were as follows: age, 18–29 years (each unit increase refers to 10 years); sex, women; hyperuricemia, no; dyslipidemia, no; hypertension, no; use of renin–angiotensin–aldosterone system (RAAS) inhibitors, no; and use of SGLT2 inhibitors, no. In ordinal logistic regression, we conducted two models according to CKD progression by eGFR and ACR categories from the 2012 Kidney Disease: Improving Global Outcomes (KDIGO) guidelines.^[17]^ In the ACR stage model, we analyzed all patients with normal eGFR (eGFR >60 mL/min/1.73 m^2^) and different levels of ACR (KDIGO stage A1–A3). In the KDIGO risk stage model, all patients were divided into four groups: stage 1, low-risk group (no renal impairment); stage 2, mildly increased risk group; stage 3, moderately increased risk group; and stage 4, high-risk group (Supplementary Table 1). Multicollinearity between variables in the logistic regression was calculated using the tolerance and variance inflation factor (VIF). The results of the tests of parallel lines showed that the two ordinal logistic regression models were statistically executable (both *P*-values >0.05). A restricted cubic spline was employed to detect a nonlinear association between DR duration and DKD development.

**Table 1:** Clinical characteristics of patients with T2DKD and T2DM

	T2DKD (*n* = 1323)	T2DM (*n* = 1351)	χ*^2^/t*	*P*
Age (years)	59.15 ± 10.86	55.54 ± 11.27	-8.42	<0.001
Gender (male)	775 (58.6%)	717 (53.1%)	8.22	0.004
BMI (kg/m^2^)	25.60 ± 3.23	25.15 ± 3.16	-3.63	<0.001
SBP (mmHg)	141.76 ± 20.54	131.72 ± 16.62	-13.91	<0.001
DBP (mmHg)	84.53 ± 11.97	81.82 ± 10.37	-6.27	<0.001
Hb (g/L)	122.13 ± 23.08	131.74 ± 16.18	12.44	<0.001
HbA1c (%)	8.81 ± 2.21	8.90 ± 2.11	1.09	0.28
BUN (mmol/L)	7.81 ± 5.23	5.45 ± 1.58	-15.69	<0.001
Scr (μmol/L)	107.60 ± 108.65	59.99 ± 13.12	-15.83	<0.001
UA (μmol/L)	297.16 ± 96.19	255.60 ± 79.06	-12.19	<0.001
Cys C (mg/L)	1.34 ± 0.95	0.83 ± 0.20	-19.08	<0.001
B2M (mg/L)	3.12 ± 2.55	1.59 ± 0.77	-21.02	<0.001
ALT (U/L)	21.79 ± 25.36	22.64 ± 19.98	0.97	0.33
AST (U/L)	19.96 ± 19.23	19.27 ± 12.09	-1.11	0.27
TP (g/L)	63.37 ± 7.85	65.34 ± 6.33	7.15	<0.001
ALB (g/L)	37.90 ± 6.44	40.88 ± 4.80	1.98	0.05
T-CHO (mmol/L)	4.65 ± 1.57	4.40 ± 1.25	-4.62	<0.001
TG (mmol/L)	2.11 ± 1.82	1.94 ± 1.62	-2.54	0.01
HDL (mmol/L)	1.09 ± 0.34	1.11 ± 0.30	1.39	0.16
LDL (mmol/L)	2.90 ± 1.17	2.74 ± 1.02	-3.86	<0.001
eGFR (mL/min/1.73 m^2^)	72.70 ± 31.53	95.28 ± 16.05	23.26	<0.001
ACR (mg/g)	149.4 (62.2–739.2)	12.0 (8.6–17.5)	-16.82	<0.001
DR	1003 (75.8%)	631 (46.7%)	126.34	<0.001
PDR^a^	327 (33.6%)	118 (18.7%)	50.04	<0.001
Duration of DR (years)	4.76 ± 2.50	3.75 ± 2.14	-15.59	<0.001
Duration of DM (years)	12.00 ± 6.91	9.78 ± 6.27	-8.68	<0.001
KDIGO risk stage				
1	NA	1351 (100%)		
2	711 (53.7%)	NA		
3	254 (19.2%)	NA		
4	358 (27.1%)	NA		
ACR stage				
1	NA	1351 (100%)		
2	690 (76.7%)	NA		
3	210 (23.3%)	NA		
Hyperuricemia	221 (16.7%)	78 (5.8%)	80.42	<0.001
Dyslipidemia	845 (63.9%)	742 (54.9%)	22.18	<0.001
Anemia	416 (31.4%)	130 (9.6%)	195.87	<0.001
Hypertension	773 (58.4%)	497 (36.8%)	99.43	<0.001
Use of RAAS				
ACEI	200 (15.1%)	109 (8.1%)	32.50	<0.001
ARB	677 (51.2%)	230 (17.0%)	347.73	<0.001
Use of SGLT2i	36 (2.7%)	52 (3.8%)	2.67	0.10

T2DKD, type 2 diabetic kidney disease; T2DM, type 2 diabetes mellitus; BMI, body mass index; SBP, systolic blood pressure; DBP, diastolic blood pressure; Hb, hemoglobin; HbA1c, hemoglobin A1c; BUN, blood urea nitrogen; Scr, serum creatinine; UA, uric acid; Cys C, cystatin C; B2M, beta-2 microglobulin; ALT, alanine aminotransferase; AST, aspartate transaminase; TP, total protein; ALB, albumin; T-CHO, total cholesterol; TG, triglyceride; HDL, high-density lipoprotein; LDL, low-density lipoprotein; eGFR, estimated glomerular filtration rate; ACR, albumin-to-creatinine ratio; DR, diabetic retinopathy; PDR, proliferative DR; DM, diabetes mellitus; KDIGO, Kidney Disease: Improving Global Outcomes; RAAS, renin–angiotensin–aldosterone system; ACEI, angiotensin-converting-enzyme inhibitor; ARB, angiotensin receptor blocker; SGLT2i, sodium–glucose cotransporter 2 inhibitor; NA, not applicable. Data are *n* (%), mean (standard deviation) or median (interquartile range), as appropriate. ^a^The proportion of PDR was calculated using the number of DR patients as the denominator.

Summary statistics for DR-associated SNPs were extracted from the results of the GWAS performed by the FinnGen consortium, which consisted of 95,752 European participants. According to the MR requirement, the SNPs that achieved genome-wide significance (*P* < 5 × 10^-8^) were selected as IV. The effects of these SNPs on the potential risk of kidney disease were assessed using summary statistics from the UK Biobank Consortium.^[18]^ A proxy SNP, which is in high linkage disequilibrium (LD; *r*^2^ > 0.8) with the target SNP, is employed when the target SNP is not available in the outcome.

To determine whether the SNPs used as IV were not in LD with each other, pairwise LD was calculated between all selected SNPs using PLINK 1.90.^[19]^ For paired SNPs that violated the independence assumption, defined as *r*^2^ > 0.01, the one with a lower *P*-value in GWAS was retained. To further ensure that the effect on exposure (DR) and on outcome (DKD) corresponded to the same allele, we harmonized the effect of these instrumental SNPs by a function that enabled all exposure and outcome alleles on the same strand. MR-Egger regression was performed to determine the presence of horizontal pleiotropic effects.^[20]^ MR estimates were calculated using the inverse variance-weighted model and the weighted median model. All analyses were conducted using the two-sample MR package in R software.^[19]^

## Results

A total of 2674 patients were included in the current study after step-by-step screening (Figure 1). The general clinical characteristics of all the patients are shown in Tables 1 and 2. Compared to the patients with no renal impairment (T2DM), those with type 2 DKD (T2DKD) were older, predominantly male, had higher SBP and DBP, and higher serum uric acid, total cholesterol, total triglyceride, and low-density lipoprotein levels. The prevalence of hyperuricemia, dyslipidemia, anemia, and hypertension was significantly higher in patients with T2DKD. The number of patients with T2DKD using ACEI, ARB, and SGLT2 inhibitors was 200 (15.1%), 677 (51.2%), and 36 (2.7%), respectively (Table 1).

Similarly, patients with DR were older, more likely to be male, and had higher serum urea nitrogen, creatinine (Cr), uric acid, cystatin C (Cys C), and beta-2 microglobulin (B2M) levels. A longer duration of diabetes and a higher prevalence of hyperuricemia, dyslipidemia, anemia, and hypertension were also found in patients with DR. The number of patients using ACEI, ARB, and SGLT2 inhibitors was 213 (13.0%), 643 (39.4%), and 58 (3.5%), respectively (Table 2).

**Table 2:** Clinical characteristics of patients with DR and non-DR

	DR (*n* = 1634)	Non-DR (*n* = 1040)	χ*^2^/t*	*P*
Age (years)	58.80 ± 10.83	55.02 ± 11.43	-8.61	<0.001
Gender (male)	934 (57.2%)	558 (53.7%)	8.22	0.004
BMI (kg/m^2^)	25.49 ± 3.23	25.17 ± 3.15	-2.54	0.01
SBP (mmHg)	138.47 ± 20.06	133.89 ± 17.63	-6.21	<0.001
DBP (mmHg)	83.20 ± 11.52	83.10 ± 10.86	-0.23	0.82
Hb (g/L)	125.31 ± 21.24	129.61 ± 18.89	5.47	<0.001
HbA1c (%)	8.77 ± 2.10	8.98 ± 2.25	2.40	0.02
BUN (mmol/L)	7.05 ± 4.54	5.93 ± 2.90	-7.77	<0.001
Scr (μmol/L)	91.08 ± 91.39	71.70 ± 57.84	-6.72	<0.001
UA (μmol/L)	283.19 ± 96.65	265.11 ± 87.20	-5.12	<0.001
Cys C (mg/L)	1.16 ± 0.82	0.96 ± 0.54	-7.86	<0.001
B2M (mg/L)	2.59 ± 2.22	1.97 ± 1.61	-8.33	<0.001
ALT (U/L)	21.69 ± 23.96	23.05 ± 20.82	1.51	0.13
AST (U/L)	19.65 ± 18.01	19.55 ± 12.29	-0.15	0.88
TP (g/L)	63.98 ± 7.46	64.96 ± 6.70	3.51	<0.001
ALB (g/L)	41.37 ± 9.90	40.15 ± 5.43	-0.40	0.69
T-CHO (mmol/L)	4.56 ± 1.54	4.47 ± 1.23	-1.79	0.07
TG (mmol/L)	2.03 ± 1.76	2.01 ± 1.66	-0.33	0.74
HDL (mmol/L)	1.09 ± 0.33	1.10 ± 0.31	0.77	0.44
LDL (mmol/L)	2.84 ± 1.13	2.79 ± 1.05	-1.02	0.31
eGFR (mL/min/1.73 m^2^)	79.82 ± 28.41	90.84 ± 24.18	10.72	<0.001
ACR (mg/g)	54.8 (14.6–339.7)	17.2 (10.03–45.5)	-9.81	<0.001
DKD	1003 (61.4%)	320 (30.8%)	238.27	<0.001
Duration of DR (years)	4.52 ± 2.46	NA		
Duration of DM (years)	12.02 ± 6.81	9.05 ± 5.94	-11.90	<0.001
KDIGO risk stage				
1	631 (38.6%)	720 (69.2%)	274.64	<0.001
2	490 (30.0%)	221 (21.3%)		
3	227 (13.9%)	27 (2.6%)		
4	286 (17.5%)	72 (6.9%)		
ACR stage				
1	631 (38.6%)	720 (69.2%)		
2	473 (28.9%)	217 (20.9%)		
3	191 (11.7%)	19 (1.8%)		
Hyperuricemia	215 (13.2%)	84 (8.1%)	16.52	<0.001
Dyslipidemia	1005 (61.5%)	582 (56.0%)	8.10	0.004
Anemia	392 (24.0%)	154 (14.8%)	32.98	<0.001
Hypertension	697 (42.7%)	365 (35.1%)	15.17	<0.001
Use of RAAS				
ACEI	213 (13.0%)	96 (9.2%)	9.00	0.003
ARB	643 (39.4%)	264 (25.4%)	55.31	<0.001
Use of SGLT2i	58 (3.5%)	30 (2.9%)	0.88	0.35

Data are *n* (%), mean (standard deviation), or median (interquartile range), as appropriate. T2DKD, type 2 diabetic kidney disease; T2DM, type 2 diabetes mellitus; BMI, body mass index; SBP, systolic blood pressure; DBP, diastolic blood pressure; Hb, hemoglobulin; HbA1c, Hemoglobin A1c; BUN, blood urea nitrogen; Scr, serum creatinine; UA, uric acid; Cys C, cystatin C; B2M, beta-2 microglobulin; ALT, alanine aminotransferase; AST, aspartate transaminase; TP, total protein; ALB, albumin; T-CHO, total cholesterol; TG, triglyceride; HDL, high-density lipoprotein; LDL, low-density lipoprotein; eGFR, estimated glomerular filtration rate; ACR, albumin-to-creatinine ratio; DKD, diabetic kidney disease; DR, diabetic retinopathy; DM, diabetes mellitus; KDIGO, Kidney Disease: Improving Global Outcomes; RAAS, renin–angiotensin–aldosterone system; ACEI, angiotensin-converting-enzyme inhibitor; ARB, angiotensin receptor blocker; SGLT2i, sodium–glucose cotransporter 2 inhibitor; NA, not applicable.

In the T2DKD group, there were 1003 patients diagnosed with DR and 337 (33.6%) were categorized as having PDR. In the T2DM group, 631 patients had DR and 118 (18.7%) had PDR (Table 1). Among those whose DR duration was less than 5 years, 127 (19.8%) and 49 (10.3%) patients had PDR in the T2DKD and T2DM groups, respectively. As mentioned in the Materials and Methods section, we divided the participants into subgroups according to the KDIGO guidelines (Supplementary Table 1). The prevalence of DR was significantly different between the groups. In patients with normal eGFR (eGFR >60 mL/min/1.73 m^2^), the prevalence of DR from the A1 to A3 subgroups was 46.7% (*n* = 631), 65.6% (*n* = 473), and 91.0% (*n* = 191), respectively. In the KDIGO risk stage 1–4 subgroup, the prevalence was 46.7% (*n* = 631), 68.9% (*n* = 490), 89.4% (*n* = 227), and 79.9% (*n* = 286), respectively (Supplementary Table 2). DR duration showed a similar pattern. Mean duration of DR in the A1–A3 subgroups was 1.93 (95% CI: 1.79–2.07), 2.68 (95% CI: 2.47–2.89), and 4.26 (95% CI: 3.93–4.59), respectively, and that in the KDIGO risk subgroups was 1.93 (95% CI: 1.79–2.07), 2.73 (95% CI: 2.52–2.93), 4.29 (95% CI: 3.97–4.60), and 4.89 (95% CI: 4.58–5.20), respectively (Supplementary Figure 1).

**Figure 2:**
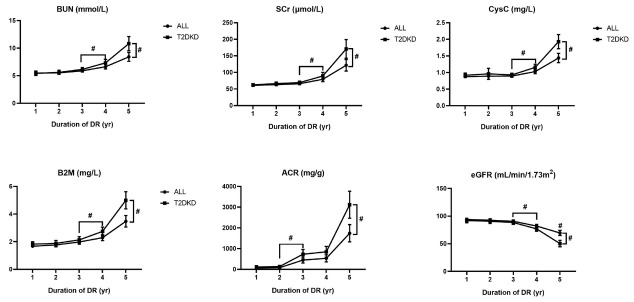
Results of renal function indicators in patients with different duration of diabetic retinopathy. Data is expressed as mean with SD. The pound sign with downward square brackets refers to significant differences between two groups with a *P*-value less than 0.05 calculated by Student’s *t*-test; the pound sign with right square brackets by the end of each line refers to significant differences among five groups with a *P*-value less than 0.05 calculated by ANOVA. ANOVA, analysis of variance; BUN, blood urea nitrogen; Scr, serum creatinine; Cys C, cystatin C; B2M, beta-2 microglobulin; ACR, albumin-to-creatinine ratio; eGFR, estimated glomerular filtration rate; DR, diabetic retinopathy; SD, standard deviation.

To explore the association between renal function and the duration of DR, we compared the serum concentrations of urea nitrogen, Cr, Cys C, B2M, eGFR, and urinary ACR in patients with DR ≤5 years. We found no statistical differences among the first 3 years, except for ACR. However, the mean concentrations of blood urea nitrogen (BUN), Cr, Cys C, and B2M were higher in the fourth-year group (6.64, 79.25, 1.03, and 2.29, respectively) and were increased significantly in the fifth-year group (8.41, 121.59, 1.44, and 3.48, respectively). In contrast, eGFR decreased significantly as the duration of DR increased (Table 3, Figure 2).

**Table 3:** Results of renal function laboratory test in patients with short-term duration of DR

	Duration of DR (years)					
Factor	1 (*n* = 190)	2 (*n* = 184)	3 (*n* = 269)	4 (*n* = 263)	5 (*n* = 212)	*P*
BUN	5.48 (1.68)	5.52 (1.60)	5.90 (2.04)	6.64 (3.49)	8.41 (5.94)	<0.001
Scr	60.88 (16.51)	63.09 (18.97)	66.00 (23.83)	79.05 (58.71)	121.59 (127.77)	<0.001
Cys C	0.88 (0.26)	0.89 (0.63)	0.89 (0.30)	1.03 (0.53)	1.44 (1.04)	<0.001
B2M	1.68 (0.77)	1.77 (0.92)	1.98 (1.34)	2.29 (1.75)	3.48 (3.09)	<0.001
eGFR	93.89 (17.93)	92.62 (17.24)	90.66 (20.03)	82.00 (25.44)	69.59 (33.56)	<0.001
ACR	62.21 (18.14)	82.10 (8.29)	449.30 (73.30)	531.40 (86.23)	1740.47 (211.41)	<0.001

ANOVA, analysis of variance; DR, diabetic retinopathy; BUN, blood urea nitrogen; Scr, serum creatinine; Cys C, cystatin C; B2M, beta-2 microglobulin; eGFR, estimated glomerular filtration rate; ACR, albumin-to-creatinine ratio. Data are expressed as mean (standard deviation). *P*-value was calculated by one-way ANOVA.

A binary and ordinal logistic regression was employed to calculate the contributions of both DR and duration of DR to the development of kidney disease in the participants. Multicollinearity between variables was not detected in any of the regression models (Supplementary Tables 3–8). In binary regression, the results showed both DR and duration of DR were significant risk factors for DKD with an adjusted OR of 3.42 (95% CI: 2.89–4.06) and 1.19 (95% CI: 1.15–1.23), respectively. Consistently, in two ordinal logistic regression models, the duration of DR was also a risk factor for ACR elevation and renal function decline. After adjusting for covariates, each year increase in duration of DR contributed to a 16% increased risk for elevation of ACR (OR = 1.16, 95% CI: 1.13–1.20) and a 21% increased risk for decline of renal function (OR = 1.21, 95% CI: 1.18–1.24; Table 4). In restricted cube spline (RCS), we found a cross-point at “2” on the X axis (duration of DR) with “1” on the Y axis (OR), which suggested that the risk for renal impairment in patients with diabetes could be increased from the duration of DR at the 2 years point (Supplementary Figure 2).

**Table 4:** Results of binary and ordinal logistic regression for DR and DKD

Regression model	Diabetic retinopathy			Duration of diabetic retinopathy		
	β-value	OR	*P*	β-value	OR	*P*
Diabetic kidney disease (binary)						
Model 1	1.27 (0.08)	3.58 (3.03–4.22)	<0.001	0.21 (0.02)	1.23 (1.20–1.27)	<0.001
Model 2	1.19 (0.09)	3.30 (2.79–3.90)	<0.001	0.19 (0.02)	1.21 (1.18–1.25)	<0.001
Model 3	1.23 (0.09)	3.42 (2.89–4.06)	<0.001	0.17 (0.02)	1.19 (1.15–1.23)	<0.001
Model 4	1.22 (0.09)	3.39 (2.84–4.02)	<0.001	0.19 (0.02)	1.21 (1.18–1.25)	<0.001
ACR stage (ordinal)						
Model 1	1.23 (0.41)	3.43 (1.54–7.69)	0.003	0.16 (0.05)	1.18 (1.07–1.30)	<0.001
Model 2	1.21 (0.14)	3.35 (2.57–4.37)	<0.001	0.16 (0.02)	1.17 (1.14–1.21)	<0.001
Model 3	1.20 (0.11)	3.33 (2.71–4.11)	<0.001	0.15 (0.02)	1.16 (1.13–1.20)	<0.001
Model 4	1.19 (0.10)	3.29 (2.73–3.97)	<0.001	0.15 (0.02)	1.16 (1.12–1.19)	<0.001
KDIGO risk stage (ordinal)						
Model 1	1.29 (0.26)	3.65 (2.21–6.04)	<0.001	0.24 (0.04)	1.27 (1.17–1.38)	<0.001
Model 2	1.20 (0.13)	3.33 (2.58–4.31)	<0.001	0.22 (0.01)	1.24 (1.21–1.28)	<0.001
Model 3	1.18 (0.10)	3.24 (2.67–3.93)	<0.001	0.19 (0.01)	1.21 (1.18–1.24)	<0.001
Model 4	1.21 (0.08)	3.36 (2.85–3.96)	<0.001	0.22 (0.01)	1.25 (1.22–1.28)	<0.001

DR, diabetic retinopathy; DKD, diabetic kidney disease; RAAS, renin–angiotensin–aldosterone system; SGLT2, sodium–glucose cotransporter 2; OR, odds ratio; CI, confidence interval; KDIGO, Kidney Disease: Improving Global Outcomes; ACR, albumin-to-creatinine ratio. Data are expressed as β-value (standard error) and OR (95% CI). Model 1 was calculated by only diabetic retinopathy or duration of diabetic retinopathy without other covariates. Model 2 was calculated adjusting age and gender. Model 3 was multivariable logistic regression including age, gender, hyperuricemia, dyslipidemia, and hypertension. Model 4 was multivariable logistic regression including age, gender, use of RAAS and SGLT2 inhibitors.

**Figure 3:**
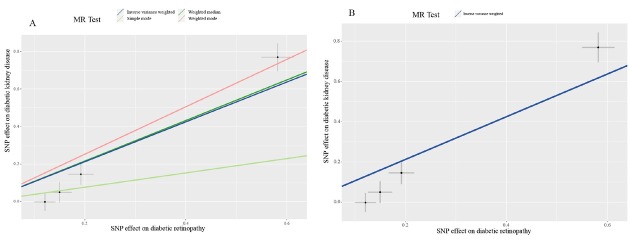
Mendelian randomization scatterplot for diabetic retinopathy on diabetic kidney disease analysis. The dark blue line shows the result of inverse variance weighting model (A and B) and the dark green line, light green line, and light red line show the results of weighted median model, simple model, and weighted model, respectively.

In the two-sample MR analyses, after implementing the pruning strategy described in the Materials and Methods section, four LD-independent SNPs that achieved genome-wide significance for DR in the FinnGen consortium were extracted: rs10490924, rs10737680, rs2596560, and rs9275207. All of these remained for performance of MR analyses of kidney disease traits. The results of MR-Egger regression showed that the estimated value for the intercept term was null for DR and DKD (β = –0.19, 95% CI: –0.39–0.001, *P* = 0.06), suggesting that there was no horizontal pleiotropy effect. MR estimates, using the inverse variance weighted (IVW) model, demonstrated that having DR was casually associated with the development of DKD with an OR of 2.89 (95% CI: 1.76–4.75; Table 5). The slope estimates of the other methods, including the simple model, weight median model, and weight model, were consistent with the IVW model (Table 5, Figure 3). We then searched the GWAS Catalog, PDGENE, and NCBI database to verify the biological functions of the SNPs involved in this study. The results indicated that none of these SNPs were directly associated with renal function, which supported the robustness of our MR analyses (Supplementary Table 9).

**Table 5:** Results from different MR methods for the effects of DR on DKD

Method	nSNP	β (95% CI)	OR (95% CI)	*P*
Inverse variance weighted	4	1.06 (0.56–1.56)	2.89 (1.76–4.75)	<0.001
MR-Egger	4	1.66 (1.31–2.02)	5.29 (3.69–7.57)	0.01
Weighted median	4	1.08 (0.80–1.36)	2.95 (2.23–3.90)	<0.001
Weighted model	4	1.26 (0.98–1.55)	3.54 (2.67–4.69)	0.003

DR, diabetic retinopathy; DKD, diabetic kidney disease, CI, confidence interval, OR, odds ratio; MR, magnetic resonance.

## Discussion

The high prevalence of renal impairment in people with diabetes is a serious concern. According to our previous cross-sectional survey, 35.5% of rural participants and 48.0% of urban participants had renal impairment among those with diabetes.^[9, 22]^ The promotion of routine community health screening is an urgent and essential issue. However, there are several limitations to kidney disease screening for residents living in economically disadvantaged areas. For example, storage, transportation, and laboratory tests for urinary and blood samples are invasive, expensive, and difficult to repeat in a short time period. On the other hand, fundoscopy is inexpensive, noninvasive, and easy to perform without extra restrictions such as overnight fasting.

Along with the increasing calculation capability of machine learning approaches, especially the development of deep-learning models, fundus images can provide various types of data that manifest the variations in microvascular structure, detect microaneurysms, and so on. Studies have also reported that changes in the retinal vasculature are associated with renal impairment.^[23, 24, 25]^ Combined with personal disease history and deep-learning models, retinal fundus images could be valuable in predicting chronic kidney impairments in patients with diabetes. In our current study, we found that the prevalence of DR increased with a decline in renal function in patients with diabetes. The duration of DR was related to ACR and eGFR progression. After adjusting for multiple variables, both DR and duration of DR were found to be significant risk factors for the development and progression of DKD. The RCS implied that short-term duration of DR could contribute to a higher risk of DKD. The MR analyses further demonstrated the causal effects of DR and DKD.

Having a long-term duration of DR, over 10 years, is believed to be an independent risk factor for renal dysfunction in patients with diabetes. However, by analyzing patients with DR ≤5 years, we found that the serum concentrations of urea nitrogen, Cr, Cys C, and B2M were much higher in the 5-year duration group than in the 1–4-year groups. The RCS claimed that the OR for the development of DKD rose to higher than 1 since the duration of DR was over 2 years. Both binary and ordinal logistic regression indicated a 19% and 21% risk of renal impairment, respectively, with an increase in the 1-year duration of DR. These results suggest that short-term DR may be associated with renal impairment. This association is supported by several findings. The Cardiovascular Health Study (CHS) analyzed the retinal and serum Cr data of 1394 participants and found that retinopathy was associated with early signs of renal dysfunction. In CHS, patients with retinopathy showed significantly elevated Cr and decreased eGFR during the 4-year study period.^[26]^ Ooi *et al*.^[27]^ and Gu *et al*.^[28]^ found that retinal vascular diameter could discriminate between early- and late-stage CKD, especially in patients with diabetes. Another cohort study reported an association between a wider central retinal vein equivalent and incident CKD in patients with diabetes during a 20-year follow-up.^[29]^

Furthermore, Cys C, a sensitive biomarker for CKD, has also been found in the retinal pigment epithelium.^[30]^ Several studies have indicated that Cys C is associated with age-related macular degeneration by inhibiting cathepsins and is an independent risk factor for DR.^[31, 32, 33]^ In the current study, we also found that serum Cys C concentration was higher in patients with DR and increased in the fifth year of DR. These results suggest that lesions in the retinal vasculature of individuals with diabetes are associated with renal dysfunction and could be a potentially sensitive biomarker for early-stage renal disease.

By performing this two-sample MR analysis using summary statistics for DR and DKD in a large sample population, we demonstrated that DR was causally associated with the development of DKD. This result was consistent with previous observational studies.^[27, 28, 29]^ The main mechanisms contributing to DKD are microvascular complications of diabetes, which are also found in many retinal diseases.^[34]^ The RAAS is found in the renal and retinal components. Angiotensin II (Ang II) not only induces endothelial dysfunction and inflammatory response by producing reactive oxygen species (ROS), but also participates in regulating the gene expression, activating intracellular signaling pathways, and remodeling the extracellular matrix.^[35]^ A previous study reported that increased levels of renin, prorenin, and Ang II were found in eyes with DR.^[36]^ The rs2596560 SNP used in the current MR analyses was found to be associated with adrenocortical complications (*P* = 2.00 × 10^-9^) and diabetes (*P* = 1.50 × 10^-9^). The UK Biobank further confirmed its association with the human leukocyte antigen (HLA) region.^[37]^ The rs 10737680 is also reported to be associated with interleukin-6 receptor.^[38]^ These results may provide evidence for the causal role of DR in the development of DKD, since inflammatory factors cause lesions in renal function in patients with DR, while the effects of confounding factors are minimized.

Our study has several limitations. First, this was a single-center retrospective study. The lifestyle, dietary patterns, and other corresponding factors of the patients might be similar. Second, our MR analyses were conducted using a European rather than an Asian population. There are several explanations for this. First, there was a lack of genome databases that analyzed DR or DKD in a large Asian population. Second, rs10490924 and rs 10737680 have been demonstrated to be associated with eye diseases in both European and Asian populations.^[39, 40, 41]^ In addition, according to the 1000 Genomes Consortium, the frequencies of allele effects of rs2596560 (T) and rs9275207 (G) are reported to be similar in European and Asian populations.^[42]^ These results suggest that our MR analyses are capable of being replicated in Asian populations.

In conclusion, we found that both DR and the duration of DR were independent risk factors for the development and progression of DKD. The short-term duration of DR may be associated with DKD development. DR had a statistically significant effect on DKD.

## Supplementary Material

Supplementary material
