# Supplementary material for: Short-term duration of diabetic retinopathy as a predictor for development of diabetic kidney disease
Source: J Transl Int Med. 2023 Dec 20;11(4):449–58. doi: 10.2478/jtim-2022-0074 (PMC10732346; doi:10.2478/jtim-2022-0074)
Supplement: Supplementary file 1 — Supplementary material [file jtim-2022-0074_sm.pdf]

Supplementary Material

Supplementary Table 1: Subject number in different KDIGO stages

		ACR (mg/g)		
	eGFR (mL/min/1.73m^2)	< 30	30 – 300	> 300
CKD 1	≥ 90	872	402	91
CKD 2	89 – 60	469	298	119
CKD 3a	59 – 45	21	40	76
CKD 3b	44 – 30	8	25	74
CKD 4	29 – 15	0	17	83
CKD 5	< 15	0	10	69

ACR stage, A1 = eGFR > 60 and ACR < 30; A2 = eGFR > 60 and ACR = 30 – 300; A3 = eGFR > 60 and ACR > 30; KDIGO risk stage, low risk = eGFR ≥ 60 and ACR < 30; mildly increased risk = eGFR ≥ 60 and ACR = 30 – 300, or eGFR = 45 – 59 and ACR < 30; moderately increased risk = eGFR ≥ 60 and ACR > 300, or eGFR = 45 – 59 and ACR = 30 – 300, or eGFR = 30 – 44 and ACR < 30; high-risk = eGFR < 30 and ACR < 30, or eGFR < 45 and ACR = 30 – 300, or eGFR < 60 and ACR > 300. eGFR: estimated glomerular filtration rate; ACR: albumin-to-creatinine ratio; KDIGO: kidney disease: improving global outcomes; CKD: chronic kidney disease.

Supplementary Table 2: Prevalence of diabetic retinopathy in different stages

Stage	KDIGO Risk (*n* = 2674)	ACR (*n* = 2251)
1	46.7% (44.0%–49.4%)	46.7% (44.0%–49.4%)
2	68.9% (65.5%–72.3%)	65.6% (61.8%–69.3%)
3	89.4% (85.6%–93.2%)	91.0% (87.0%–94.9%)
4	79.9% (75.7%–84.1%)	NA
P_{trend}	< 0.001	< 0.001

Data was expressed as prevalence (95% confidence interval). P_{trend} was calculated by Mantel-Haenszel chi-square test. NA, not applicable. ACR: albumin-to-creatinine ratio; KDIGO: kidney disease: improving global outcomes.

Supplementary Table 3: Multicollinearity test of binary logistic regression for DR and DKD

Variables	Tolerance	VIF
Age	0.94	1.06
Gender	0.96	1.04
Diabetic retinopathy	0.96	1.04
Hyperuricemia	0.97	1.03
Dyslipidemia	0.96	1.05
Hypertension	0.99	1.01
RAAS	0.93	1.08
SGLT2i	0.96	1.04

Multicollinearity between variables was calculated by VIF. Multicollinearity was identified when VIF > 10 and tolerance < 0.1. DKD: diabetic kidney disease; DR: diabetic retinopathy; RAAS: renin-angiotensin-aldosterone system; SGLT2i: Sodium-glucose cotransporter 2 (SGLT2) inhibitors; VIF: variance inflation factor.

Supplementary Table 4: Multicollinearity test of binary logistic regression for duration of DR and DKD

Variables	Tolerance	VIF
Age	0.91	1.10
Gender	0.96	1.04
Duration of diabetic retinopathy	0.91	1.10
Hyperuricemia	0.96	1.04
Dyslipidemia	0.96	1.04
Hypertension	0.98	1.02
RAAS	0.93	1.08
SGLT2i	0.95	1.05

Note: Multicollinearity between variables was calculated by VIF. Multicollinearity was identified when VIF > 10 and tolerance < 0.1. DKD: diabetic kidney disease; DR: diabetic retinopathy; RAAS: renin-angiotensin-aldosterone system; SGLT2i: Sodium-glucose cotransporter 2 (SGLT2) inhibitors; VIF: variance inflation factor.

Supplementary Table 5: Multicollinearity test of ordinal logistic regression for DR and ACR stage

Variables	Tolerance	VIF
Age	0.93	1.07
Gender	0.94	1.06
Diabetic retinopathy	0.97	1.03
Hyperuricemia	0.99	1.01
Dyslipidemia	0.96	1.05
Hypertension	1.00	1.00
RAAS	0.98	1.02
SGLT2i	0.97	1.03

Note: Multicollinearity between variables was calculated by VIF. Multicollinearity was identified when VIF > 10 and tolerance < 0.1. DKD: diabetic kidney disease; DR: diabetic retinopathy; RAAS: renin-angiotensin-aldosterone system; SGLT2i: Sodium-glucose cotransporter 2 (SGLT2) inhibitors; VIF: variance inflation factor.

Supplementary Table 6: Multicollinearity test of ordinal logistic regression for duration of DR and ACR stage

Variables	Tolerance	VIF
Age	0.91	1.10
Gender	0.94	1.06
Duration of diabetic retinopathy	0.95	1.05
Hyperuricemia	0.99	1.01
Dyslipidemia	0.96	1.05
Hypertension	1.00	1.00
RAAS	0.93	1.08
SGLT2i	0.95	1.05

Note: Multicollinearity between variables was calculated by VIF. Multicollinearity was identified when VIF > 10 and tolerance < 0.1. DKD: diabetic kidney disease; DR: diabetic retinopathy; RAAS: renin-angiotensin-aldosterone system; SGLT2i: Sodium-glucose cotransporter 2 (SGLT2) inhibitors; VIF: variance inflation factor.

Supplementary Table 7: Multicollinearity test of ordinal logistic regression for DR and KDIGO risk stage

Variables	Tolerance	VIF
Age	0.94	1.06
Gender	0.96	1.04
Diabetic retinopathy	0.96	1.04
Hyperuricemia	0.97	1.03
Dyslipidemia	0.96	1.05
Hypertension	0.99	1.01
RAAS	0.95	1.05
SGLT2i	0.95	1.05

Note: Multicollinearity between variables was calculated by VIF. Multicollinearity was identified when VIF > 10 and tolerance < 0.1. DKD: diabetic kidney disease; DR: diabetic retinopathy; RAAS: renin-angiotensin-aldosterone system; SGLT2i: Sodium-glucose cotransporter 2 (SGLT2) inhibitors; VIF: variance inflation factor.

Supplementary Table 8: Multicollinearity test of ordinal logistic regression for duration of DR and KDIGO risk stage

Variables	Tolerance	VIF
Age	0.91	1.10
Gender	0.96	1.04
Duration of diabetic retinopathy	0.91	1.10
Hyperuricemia	0.96	1.04
Dyslipidemia	0.96	1.04
Hypertension	0.98	1.02
RAAS	0.94	1.06
SGLT2i	0.96	1.04

Note: Multicollinearity between variables was calculated by VIF. Multicollinearity was identified when VIF > 10 and tolerance < 0.1. DKD: diabetic kidney disease; DR: diabetic retinopathy; RAAS: renin-angiotensin-aldosterone system; SGLT2i: Sodium-glucose cotransporter 2 (SGLT2) inhibitors; VIF: variance inflation factor.

Supplementary Table 9: Correlated phenotypes of SNPs involved in MR analyses

Variant	Risk allele	Mapped gene	Phenotype	*P*
rs10490924	T	ARMS2	Age-related macular degeneration	4×10^{-322}
rs10737680	A	CFH	Age-related macular degeneration	1×10^{-434}
rs2596560	T	MICA	Adrenocortical insufficiency	2×10^{-9}
rs9275207	G	HLA-DQB1	Seropositive rheumatoid arthritis	9×10^{-34}

Phenotypes were searched on NCBI, GWAS Catalog, FINN GEN and UK Biobank database.

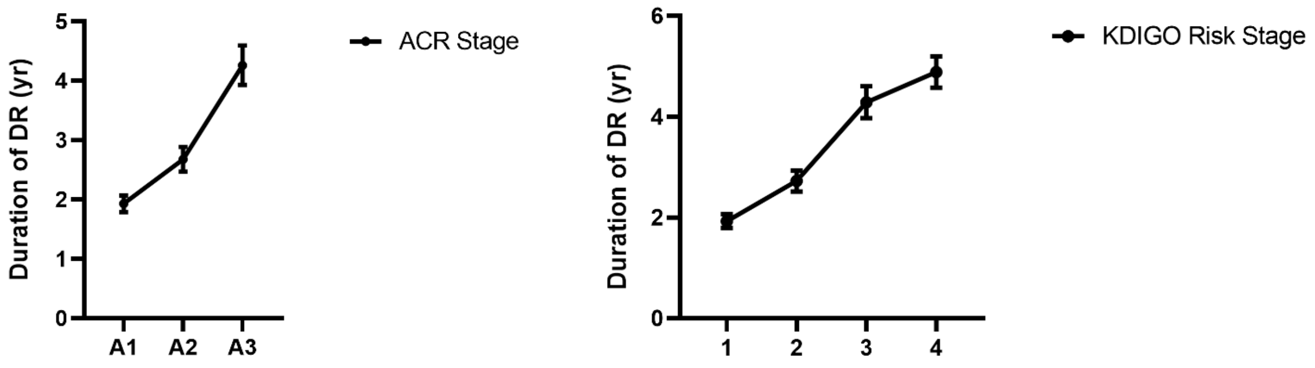

Supplementary Figure 1: Duration of diabetic retinopathy in different ACR and KDIGO risk stages. ACR: albumin-to-creatinine ratio; KDIGO: kidney disease: improving global outcomes.

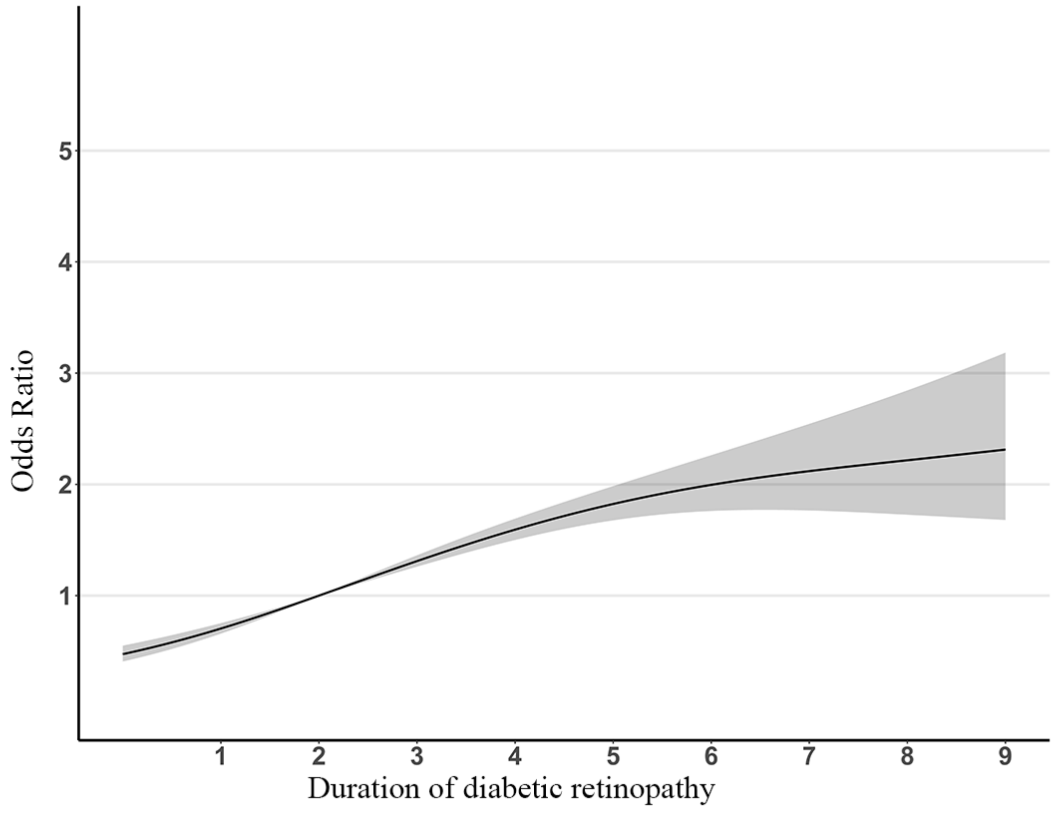

Supplementary Figure 2: Restricted cube spline for duration of diabetic retinopathy and odds ratios of development of diabetic kidney disease. The four-node spline method was employed in current study.
